# The Use of Alfa-Lipoic Acid-R (ALA-R) in Patients with Mild-Moderate Carpal Tunnel Syndrome: A Randomised Controlled Open Label Prospective Study

**DOI:** 10.5704/MOJ.2003.001

**Published:** 2020-03

**Authors:** M Passiatore, A Perna, R De-Vitis, G Taccardo

**Affiliations:** 1 Department of Orthopaedics and Traumatology, Fondazione Policlinico Universitario A. Gemelli IRCCS, Rome, Italy; 2 Department of Orthopaedics and Traumatology, Universita Cattolica del Sacro Cuore, Rome, Italy

**Keywords:** carpal tunnel syndrome, alpha-lipoic acid, ALA-R, neuroprotection, median nerve compression

## Abstract

**Introduction:**

Carpal tunnel syndrome is one of the most common peripheral neuropathies. Only a few studies evaluate the efficacy of “nutraceuticals” on peripheral nerves and neuropathic pain. The aim of the present investigation is to evaluate the role of Alfa-Lipoic Acid-R (ALA-R) on clinical and functional outcomes in patients affected by mild to moderate carpal tunnel syndrome.

**Material and Methods:**

The present investigation is a prospective randomised controlled open label study, performed at our Hand Surgery Department (Fondazione Policlinico Universitario A. Gemelli IRCCS, Rome) from October 2018 to March 2019. The enrolled patients were divided in two groups: Group A (ALA-R 600mg once day for 60 days) and Group B (control Group, no drug administration).

**Results:**

134 patients (74 F, 60 M) met the inclusion and exclusion criteria. In Group A, there was a statistically significant pain reduction compared to the control Group. Using the Boston Carpal Tunnel Questionnaire, there were no significant improvements in the other symptoms and function.

**Conclusion:**

ALA-R full dose administration for two months leads to positive short term results in terms of symptoms and function improvement, even if the surgical carpal tunnel release remains the treatment of choice.

## Introduction

Carpal Tunnel Syndrome (CTS) is one of the most frequent and disabling peripheral nerve compression syndromes^1, 2^. Incidence of CTS is increasing^3^, and the request for treatment is increasing as well^4, 5^.

The increased pressure into the carpal tunnel reduces the blood flow to median nerve^6^, and many risk factors and comorbidities can contribute to it^7^. Chronic endoneural ischemia increases the local oxidative stress, resulting in degenerative changes in the nerve^8^. Even if the surgical carpal tunnel release can definitely reduce symptoms^9^, many non-surgical treatments have been proposed^10^–^12^, to address the growing request for further treatments.

Alfa-Lipoic Acid (ALA) is a well-known molecule, and its biochemical and metabolic features have been described in oxidative stress models like diabetes^13, 14^. The effect of ALA in CTS has been investigated^15^–^18^.

Synthetic ALA is produced as a racemic mix^15^, and the more active enantiomer is the Alfa-Lipoic Acid-R (ALA-R), the dextrorotatory one^19^. No study has investigated before the effects of ALA-R on CTS at the maximum dosage recommended (600mg per day). The aim of the present study was to investigate prospectively the role of high dosage ALA-R therapy on clinical and functional outcomes in patient affected by mild-moderate carpal tunnel syndrome, who have to undergo surgical treatment.

## Materials and Methods

The present investigation is a one-centre prospective randomised controlled open label prospective study, performed at our Hand Surgery Department, Fondazione Policlinico Universitario A. Gemelli IRCCS in Rome, from October 2018 to March 2019.

One hundred and third-four patients who were listed for open surgical carpal tunnel release, were enrolled for this study. Written informed consent, was taken from all patients before the enrolment in the study. All patients enrolled were informed that the surgical carpal tunnel release, was the necessary treatment for CTS, and that a pharmacological therapy could not replace the beneficial effects of a surgical operation to date.

Inclusion and exclusion criteria are shown in Table I. All patients who are older than 18 years old, with an electromyographically confirmed diagnosis of mild-moderate mono or bilateral CTS, according with classification of Padua *et al*^20^, with typical CTS symptoms (positive Phalen maneuvers and Tinel sign and paraesthesia in the median nerve region) were eligible for the study. The results were assessed only on the dominant hand.

**Table I:** Inclusion/exclusion criteria

Inclusion Criteria	Exclusion Criteria
Age: more than 18 years old Moderate mono or bilateral CTS, according with classification of Padua *et al*^20^ and contemporary presence of typical CTS symptoms (positive Phalen maneuvers and Tinel sign and paresthesia in the median nerve region).	Diabetes Neuromuscular disease Hepatic impairment (MELD Score > 9) Moderate to severe renal impairment (creatinine clearance < 90 ml/min) Known psychiatric disorders Allergy or contraindication Pregnancy Breastfeeding Concomitant use of other neuroprotective drugs or nutraceutical substances

Patients who suffered from diabetes, neuromuscular disease, hepatic impairment (MELD Score > 9), moderate to severe renal impairment (creatinine clearance < 90ml/min), known psychiatric disorders, allergy or contraindication to the study drugs were excluded from the study. Pregnant and breastfeeding women were excluded too. Patients with history of trauma or previous surgery in their dominant hand side were excluded. Patients who reported concomitant use of other neuroprotective drugs or nutraceutical substances during follow-up visit were excluded from the study.

The study was in accordance with the national ethics criteria. The study was also in accordance with the Helsinki convention and Good Clinical Practice. Considering that the administration of ALA-R was already used in the preoperative management protocols at our institute, a formal ethical approval was not requested for this study.

**Fig. 1:**
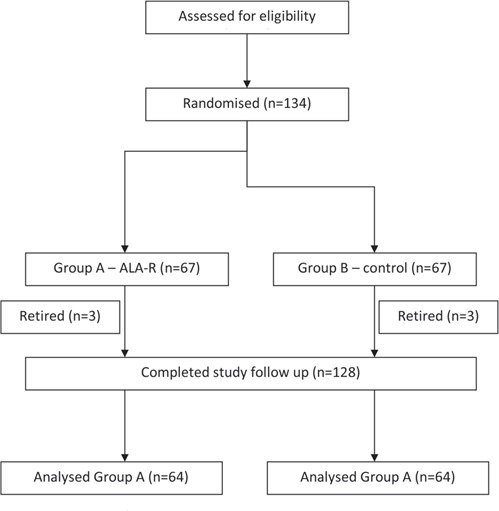
Study flow chart.

After the enrolment in the study, patients were randomly assigned to the two groups studied with a 1:1 allocation ratio. Randomisation process was performed in blocks of 10. The randomisation scheme was generated by using the Web site Randomization.com (http://www.randomization.com) (Fig. 1).

The enrolled patients were divided into two groups: Group A (Alfa Lipoic Acid-R (ALA-R) 600mg once day for 60 days) and Group B (control group, no drug administration).

During the first visit, the informed consent, demographic data (age, sex, BMI), medical history, and electromyographic data were collected. Pain was evaluated using the visual analogue scale (VAS). The patient symptoms and the functionality of the affected hand were evaluated using the Boston Carpal Tunnel Questionnaire (BCTQ).

During the second visit, two months after the enrolment, pain and functionality were evaluated again. Patients were asked about the two items: pain and functionality, both day and night.

The aim of the study was to evaluate the effect of ALA-R in the management of patients with mild-moderate carpal tunnel syndrome. The primary outcome was the evaluation of patients’ symptoms and functionality using the BCTQ. Secondary outcomes were: pain reduction measured with the VAS (both day and night), side effects, and the general satisfaction.

The target sample size was 120, with 60 patients in each group, considering an effect of 0.7 unit reduction in BCTQ value, with 80% power and 5% probability of type 1 error and assuming a standard deviation of 1.50 for this parameter. Considering a 10% possible dropout rate, 67 patients per group or 134 patients overall were recruited.

The BCTQ scores were ordinal variables used as continuous variables according to other authors^21^. Non-parametric tests were used. Wilcoxon signed rank test (paired analysis) was used to compare the variation of pain and BCTQ values between t0 and t1. Mann-Whitney U test was used to compare the final assessment results between group A and B (independent samples).

The significance was established for a value of p<0.05. Dedicated SPSS (version 20.0.0) statistical calculation software (SPSS Inc, Chicago, IL) was employed. Data were described using means and standard deviations for quantitative variables and numbers and percentages for qualitative variable. Only one decimal digit was reported and was rounded up.

## Results

One hundred and thirty-fourth patients (74 F, 60 M) who met the inclusion and exclusion criteria were enrolled in the study. Ninety (67.2%) patients were right-handers. Group A included 67 patients (38 F, 29 M); Group B (control group) included 67 (37 F, 30 M) patients who had not received pharmacological treatments.

The mean age in Group A was 66 years old (+/-10.5), in Group B was 69 years old (+/-11.3). The mean BMI was 26.5 (+/-2.9) in Group A, 28.0 (+/-3.9) in Group B. In Group A 18 (26,9%) patients were smokers, in Group B 17 (25.4%). A summary of the characteristics of the patients enrolled in the study is reported in Table II.

**Table II:** Clinical and demographic features

Demographics	Group A	Group B
Number of patients	67	67
Gender	38 F, 29 M	37 F, 30 M
Age (years)	66.1 (+/-10,5)	69.0 (+/-11.3)
BMI	26.5 (+/-2,9)	28.0 (+/-3,9)
Symptom duration (months)	18.4 +/- 5,0	20.0 +/- 4,5
Smokers	18 (26,9%)	17 (25,4%)
History of chronic alcohol consumption	3 (4,5%)	2 (2,9%)
Comorbidities with impact on peripheral nervous system*	10 (14.9%)	11 (16.4%)
Other comorbidities**	22 (32,8%)	22 (32,8%)

*cervical spine disease, rheumatoid arthritis

**hypercholesterolemia, hypertension, glaucoma, history of cardiac infarction

Three patients (4.5%) in Group A did not complete the therapy cycle because of side effects (one patient reported headache, two reported nausea) and they were excluded from the study. There were no patients lost at follow-up in Group A. Three patients were lost (4.5%) in Group B. Concerning BTCQ, after two months, scores improved from 3.5 (+/-1.3) to 3.0 (+/-1.0) (p=0.072) in Group A. In Group B did not change significantly, from 3.8 (+/-1.4) to 3.9 (+/-1.5) (p=0.270). Comparing the final assessment results between group A and B (t1), Mann Whitney U-test showed no statistically significant difference (p=0.194) (Table III).

**Table III:** The results. Data represent the mean +/- SD

	ALA-R (Group A)			Control (Group B)			t1
	t0	t1	p value	t0	t1	p value	(Group A vs Group B)
BTCQ score	3.5 (+/-1.3)	3.0 (+/-1.0)	0.072	3.8 (+/-1.4)	3.9 (+/-1.5)	0.270	0.194
Pain night (VAS score)	6.0 (+/- 1.5)	2.9 (+/-1.3)	<0.0001	6.3 (+/- 0.8)	6.5 (+/-1.3)	0.232	<0.0001
Pain day (VAS score)	5.3 (+/- 1.4)	1.9 (+/-1.3)	<0.0001	6.5 (+/- 1.2)	6.6 (+/-1.3)	0.200	<0.0001

Pain at night; in Group A the mean VAS score at t0 was 6.0 (+/- 1.5), decreased to 2.9 (+/-1.3) at two months, (p<0.0001). In Group B the mean VAS score at t0 was 6.3 (+/- 0.8), and increased to 6.5 (+/-1.3) at two months (p=0.232). Comparing the final assessment results between group A and B (t1), Mann Whitney U-test showed statistically significant differences (p<0.0001) (Table III).

Regarding pain during the day, in Group A the mean VAS score at t0 was 5.3 (+/- 1.4), decreased to 1.9 (+/-1.3) at two months, (p<0.0001). In Group B the mean VAS score at t0 was 6.5 (+/- 1.2), and increased to 6.6 (+/-1.3) at two months (p=0.200). Comparing the final assessment results between group A and B (t1), Mann Whitney U-test showed statistically significant differences (p<0.0001) (Table III).

After two months (the last follow-up), at the question “how are you with your disease (CTS)?”, in Group A, 9 patients (14.0% of the patients who completed the follow-up) declared that they do not need further treatment at the moment, because of the satisfying relief of symptoms. In Group B all patients asked to be treated.

## Discussion

Other studies had already investigated the efficacy of nonsurgical treatment of CTS, sometimes with promising short term results^22, 23^. However, even if the results supporting the conservative treatment were not straightforward^24^, conservative treatment could be effective as well, and is therefore recommended for patients with very low grade CTS^17, 25^. The efficacy of ALA in CTS has been already investigated. Pajardi *et al* prospectively investigated the efficacy of a mix of natural substances including ALA administered from three months before surgery for CTS, followed up to three months after surgery. They observed that taking therapy both before and after surgery improved nocturnal symptoms and Phalen test result. Differently from what we did, they evaluated patients only after surgery. Furthermore, they did not use exclusively ALA-R, and they administered ALA 300mg twice a day. Hence, the positive results they obtained came from different factors^15^.

Similarly, another more recent prospective study had investigated the effect of ALA before and after surgery in CTS, they only assessed post-surgical improvement^18^. Luchetti *et al* reported short term results (two months follow-up) from an observational Italian multicentric study about conservative treatment of CTS. They studied the effects of conservative treatment in patients with a CTS diagnosis (from low to extremely severe). At the end of follow-up, less than 50% of patients were scheduled for surgery. Their results suggested a use of a conservative therapy based on neurotrophic agents, especially ALA, in order to control symptoms (both nocturnal and diurnal pain) and improve function^17^. Other studies demonstrated the efficacy of a combination of ALA and gamma-linolenic acid in up to severe CTS^16, 18^. All the above mentioned studies^15-18^ investigated specifically ALA use in CTS, but they did not distinguish between the ALA-R and racemic preparations.

Conversely, we considered this distinction to be important. Only the ALA-R exists in nature and it works as a cofactor in many chemical reactions^19^. Currently, ALA-R is considered the eutomer^26^, and different preparations of the drug have been described^27, 28^. Both enantiomers are equally absorbed in proportion to the dose administered from 50 to 600mg (Time of Maximum Concentration: from 30 to 60 minutes)^29^, but they are poorly bioavailable (less than 30%)^30^. Food intake further decrease bioavailability^31^. In view of all this, evidence to date indicates how to optimise the use of ALA-R^14^, and it should not be overlooked.

Concerning our study, although the BCTQ score (primary outcome) was reduced in Group A compared to the control group, the difference between the two groups was not statistically significant. Furthermore, there was no functional improvement comparing t0 and t1 in each group. However, our data show that the use of ALA-R had a favorable impact on pain, both night and day.

Therefore the administration of ALA-R full dose could be effective in reduction of pain in patients who are waiting for surgical treatment. None had prospectively evaluated before how a full “one-shot” dose of the eutomer (ALA-R) could impact in terms of symptoms improvement in CTS. To the best of our knowledge, the biochemical activity of ALA could not stop the process of nerve degeneration of the progressing disease^14, 15, 32^. Similarly, evidence to date suggested that conservative treatment alone did not result in “complete recovery”^24^. In our institution, the waiting list for surgery could be more than 10 months long, therefore the patients could benefit from the administration of the ALA-R pending surgery. Furthermore, patients who could not undergo surgery (because of major health problems) or who had to postpone surgery, or who did not undergo surgery for personal convictions could benefit from our non-surgical treatment.

This study had the limitation of a small follow-up. It was possible that patients could relapse with symptoms again after treatment. Nevertheless it was equally possible that a second period of treatment could relieve symptoms again. Further studies should be done. Longer follow-up should be considered to evaluate the maintenance of beneficial effects, and to assess the possibility to repeat the drug therapy.

## Conclusion

ALA-R is a safe drug. No major side effects were observed in our study. Compliance results were positive, probably because therapy was simply taken once a day. ALA-R full dose administration for two months led to positive short term results in terms of pain improvement. Even if the surgical carpal tunnel release is the treatment of choice, all patients with a clear diagnosis of CTS should be treated with ALA-R if not contraindicated. Patients should be correctly informed about the uncertain and non-durable outcome of this treatment.
